# Development, reliability and validity of the Chichewa WHOQOL-BREF in adults in lilongwe, Malawi

**DOI:** 10.1186/1756-0500-5-346

**Published:** 2012-07-03

**Authors:** Tim Colbourn, Gibson Masache, Jolene Skordis-Worrall

**Affiliations:** 1 Parent and Child Health Initiative (PACHI), Amina House (top floor, Entrance 3), Paul Kagame Road roundabout, P.O. Box 31686, Lilongwe 3, Malawi; 2 UCL Centre for International Health and Development, 30 Guilford Street, London, WC1N 1EH, UK

**Keywords:** Quality of life, Malawi, Translations, Validation studies, WHOQOL-BREF

## Abstract

**Background:**

Quality of life measurement is a useful addition to measurement of health outcomes in evaluation of the benefits of many health and welfare interventions. The WHOQOL-BREF measures quality of life from a broad multi-dimensional perspective but was not used in Malawi. The objective of this study was to translate the WHOQOL-BREF questionnaire into the main local language of Malawi: Chichewa; and to validate it quantitatively with respect to internal consistency, domain structure, and discriminant validity for this context.

**Methods:**

WHO-mandated guidelines were followed for translation, adaptation, pre-testing (focus groups), piloting (patient interviews) and data coding. Analyses using descriptive statistics, correlation and regression were undertaken to investigate the validity of the WHOQOL-BREF in the ways described above. Additional regression analyses examined the impact of socio-demographic variables on the domain scores.

**Results:**

309 respondents completed the questionnaire (with >98% response rates for all questions except Q21 (sex life)). 259 were sick with a variety of health problems, and 50 were considered healthy. All domains showed adequate internal consistency (Cronbach’s alpha > =0.7) with all item scores also most highly correlated with the scores of their assigned domain. All domain scores varied by health problem, and more depressed respondents had significantly lower scores in all domains than those less depressed. Domain scores and their associations with socio-demographic variables are presented and discussed.

**Conclusion:**

This study demonstrates that the new Chichewa WHOQOL-BREF questionnaire is acceptable and comprehensible to respondents in Malawi. The questionnaire also passed a number of tests of the validity of its psychometric properties. In the pilot population we found that older age was associated with lower Physical domain scores. Conversely, higher levels of educational attainment were found to be associated with higher quality of life in all domains except for Social Relationships. Respondents living as married or single were found to have higher quality of life in the Physical, Psychological and Social domains, and those who were widowed lower Physical quality of life.

## Background

Measurement of overall Quality of Life is a useful addition to measurement of mortality and morbidity in evaluation of the benefits of many health and welfare interventions [1]. In the last decade, efforts to measure overall quality of life have increased and measures such as the WHOQOL-100 have been constructed and cross-culturally validated [2]. The WHOQOL-BREF is the shorter version of this questionnaire. Although it only has 26 questions as opposed to 100, it aims to cover a broad range of quality of life facets, divided into four domains: Physical; Psychological; Social, and Environmental [3].

The WHOQOL-BREF has so far been translated and validated for use in many countries [3] (for specific examples of individual countries see e.g. [4-7]) but has not yet been validated in Malawi. Chichewa is the dominant spoken and written language and national language of Malawi, spoken by around two-thirds of the population, and understood by nearly all Malawians, especially in the populous central and southern regions [8]. Chichewa is also spoken in parts of Zambia, Zimbabwe and Mozambique [8]. This study describes the translation from English to Chichewa, adaptation, and piloting process that constitutes the validation of the WHOQOL-BREF in Malawi. The focus of this paper is on the results of the quantitative validation.

Following this foundation it is hoped that the Chichewa version of the WHOQOL-BREF will be used more frequently to evaluate the impact of a wide range of health, welfare and other interventions in the Malawian context.

## Methods

Throughout the translation, adaptation, and piloting of the translated survey instrument we endeavoured to follow the protocol as set out in the WHOQOL user manual [9] and WHO Translation method [10]. In this paper we focus on the quantitative validation of the questionnaire. This study took place between September 2009 and April 2010.

## Ethics

We complied fully with ethical principles in this study and ethical approval was granted for this study both in Malawi by the NHSRC (Protocol #696) and in the UK by UCL (Project ID: 2105/001). All respondents gave informed consent prior to being included in the study and were aware that they could suspend participation in the study at any time.

### Translation, adaptation and pre-testing

The English version of the WHOQOL-BREF was first translated into the main local language of Malawi, Chichewa, by four English-Chichewa bilingual individuals, as required by the WHO translation method [10].

A question asking whether you get enough food to eat was added as this was felt by the study team to be a potentially important area of quality of life in the food-insecure Malawian context. As required by the WHO, all of the 26 original questions were retained.

The initial Chichewa version was pre-tested on a group of monolingual Chichewa speakers with little formal education, who reflected on the questions, finding them to be generally comprehensible with only minor adaptations required. The bilingual translation group then undertook the suggested adaptations to the original wording.

The revised Chichewa document was then back-translated into English by a professional translator. The original English version was compared to the re-translated document. A few significant differences were found and these were re-translated and compared by the bilingual group until equivalence in both languages was achieved as far as possible. A few problems with equivalence remained as described below:

• Abstract words, which often do not have Chichewa equivalents, were the most difficult to translate e.g. ‘standards’ (in the instructions) required the translation team to opt for descriptive phrases such as in this case: ‘mulingo omwe mumadziyika’ (‘how you measure yourself or the level you put yourself at’; which was still confused with ‘self-esteem’ in the back-translation). Another abstract phrase with which the group had problems was ‘quality of life’. There are a number of phrases to express this concept in Chichewa but most of them have another meaning different from the intended interpretation. For instance *ubwini wa moyo wanu* could also mean how beneficial is one’s life.

• Other words in English seemed to imply a specific meaning whereas, in Chichewa, the related words were more general such as the words chosen to translate ‘enjoy’ (kusangalala—to be happy), ‘concentrate’ (chidwi/chomvetsera choyenera—appropriate interest/attention), ‘safe’ (otetezedwa—protected), ‘energy’ (mphamvu—strength) and ‘physical environment’ (malo amene mumapezeka kapena kukhala kawirikawiri—the place you are found or usually stay). There is also no Chichewa word for ‘gender’.

• Some concepts could be translated but the concepts themselves are difficult to understand because of cultural differences. For example, living as married was translated perfectly well but the idea of a couple living in the same house without being married does not exist in Malawian culture. The moment a couple start living in the same house in Malawi, they are considered as being married regardless of whether they registered with the government or not, or whether they are recognized by their religion or not. We assumed that the question was trying to establish the legal status of the relationship, so we distinguished this cultural kind of ‘marriage’ (living together) from one where the couple are legally registered as married by the District Commissioner or church.

The above problems required interviewers to clarify the meaning of any perceived ambiguities with respondents as they occurred. Interviewers were trained in this process to ensure that their clarifications were accurate and consistent.

The penultimate version of the questionnaire was tested on several focus groups who discussed its content and relevance to quality of life in Malawi. Feedback from this qualitative validation of the questionnaire was incorporated into the final version of the questionnaire. The final Chichewa WHOQOL-BREF used in this pilot study is available as Additional file 1.

### Survey conduct

Piloting of the Chichewa WHOQOL-BREF needed to be undertaken among 250 people with a variety of ill-health conditions and 50 healthy people. To find such a range of people, the survey was conducted among a convenience quota sample at Kamuzu Central Hospital (KCH). KCH is the main referral hospital for the Central Region of Malawi, located in the capital Lilongwe; 259 people with various diagnoses and disabilities in a range of in-patient wards and 26 healthy respondents, often friends/relatives/guardians of the in-patients, were surveyed at KCH. A peri-urban area of Lilongwe (Area 18) and rural villages outside Lilongwe city contributed 24 healthy respondents. The survey was interviewer administered by two research assistants trained in data collection.

The mean questionnaire completion time was 19 min (standard deviation: 5 min).

Only 8 people who were approached to take part in the survey refused, giving a high response rate of 97.4 %. The majority of those who refused did not feel well enough to complete the survey.

### Analysis

All analyses presented here were done using Stata 11.2 for Mac and are reported using the wording of the original English version of the WHOQOL-BREF [9]. The SPSS syntax given in the WHOQOL user manual [9] was followed and domain scores were calculated accordingly using the formulas given:

(1) $$\begin{array}{cc} \text{Physical domain} & =\left(\left(6-\text{ Q}3\right)+\left(6-\text{Q}4\right)+\text{Q}10+\text{Q}15+\text{Q}16+\text{Q}17+\text{Q}18\right)\times 4.\\ \text{Psychological domain} & =\left(\text{Q}5+\text{Q}6+\text{Q}7+\text{Q}11+\text{Q}19+\left(6-\text{Q}26\right)\right)\times 4.\\ \text{SocialRelationshipsdomain} & =\left(\text{Q}20+\text{Q}21+\text{Q}22\right)\times 4.\\ \text{Environmentdomain} & =\left(\text{Q}8+\text{Q}9+\text{Q}12+\text{Q}13+\text{Q}14+\text{Q}23+\text{Q}24+\text{Q}25\right)\times 4. \end{array}$$

Transformation of domain score to 0–100 scale:

(2) $$\text{TRANSFORMED SCORE}=\left(\text{SCORE}-4\right)\text{ × }(100/16)$$

The internal consistency, domain structure, discriminant validity and convergent validity of the questionnaire were checked. Internal consistency was determined by calculating Cronbach’s Alpha for each of the four domains. The validity of the domain structure was determined by calculating Pearson’s Correlation Coefficients for the associations between each item (question) and each domain and determining which domain each item was most correlated too. Following others [5,11], correlations >0.45 were considered acceptable.

Socio-demographic variables and variables assessing discriminant validity were regressed against the four main domain scores and the overall quality of life, and health domain scores using OLS regression. Each variable was separately regressed first. For each domain, this was followed by testing all socio-demographic variables at once and removing p > 0.1 variables sequentially and finally removing p > 0.05 variables to arrive at restricted multivariate models. The variables assessing discriminant validity (depression (on a 1–5 scale: question 26 of the questionnaire, and disability weight on a 0 to 1 scale) were then re-tested by addition to the restricted multivariate models.

Disability weights for respondent health conditions were obtained by matching with those provided by the Global Burden of Disease 2004 study [12] (Additional file 2) and were used to assess discriminant and convergent validity of the questionnaire by regressing the weights against domain scores.

The regression analyses were not prescribed by WHO, but are considered best practice [3].

## Results

### Respondent characteristics

The sample and demographic characteristics of the respondents are provided in Table 1: 259 were sick, 50 were healthy; 61% were female, 6% had no education, 32% primary education, 45% secondary education and 17% tertiary; most were either married (59%) or single (22%) with minorities living as married (3%), separated (4%), divorced (4%) or widowed (9%); the mean age was 36.8 (std. dev.: 13.7). Respondent characteristics broadly match those of the general population as detailed in the 2004 Malawi DHS [13], except for there being less 18–25 year olds, the respondents having higher levels of education on average, and there being more widowed and less married respondents in our sample. The first two differences are likely due to the survey predominantly taking place in the central hospital in Lilongwe, a place less accessible to poorer more remote rural communities. More respondents were female because of the majority of the guardians of patients (22/26) and all 10 of those with sick children were female as is traditional in Malawi.

**Table 1:** Respondent characteristics

	**Total respondents (n = 309)**	
***Respondent characteristics***	**n**	**%**
*Category*		
Sick (KCH)	259	83.8 %
Healthy (KCH)	26	8.4 %
Healthy (village)	24	7.8 %
*Gender*		
male	121	39.2 %
female	188	60.8 %
*Education*		
None	18	5.8 %
Primary	99	32.0 %
Secondary	139	45.0 %
Tertiary	53	17.2 %
*Marital Status*		
Single	69	22.3 %
Married	181	58.6 %
Living as Married	8	2.6 %
Separated	11	3.6 %
Divorced	13	4.2 %
Widowed	27	8.7 %
*Age*	mean	36.8
	std. dev.	13.7
	min.	17
	max.	80

As stipulated in the criteria for piloting the WHOQOL-BREF [9], the respondents interviewed had a wide range of illnesses and health problems; corresponding to a wide range of disability weights (Additional file 2).

### Facet scores

Additional file 3: Table S1 presents the frequencies, distributions and averages of the responses to each of the 27 facets (questions) included in the final Chichewa version of the WHOQOL-BREF. Question 1 constitutes a separate domain of the WHOQOL-BREF which seeks to measure overall quality of life [9]. Question 2 is also considered as a separate domain which is intended to measure overall health-related quality of life [9]. The other questions map onto the four domains as detailed in Table 2 (discussed below).

**Table 2:** Floor and ceiling effects of items on domain scores

	**Floor effects**		**Ceiling effects**	
	**%**	**Rank**	**%**	**Rank**
**Physical (item no.)**				
Physical Pain (3)	5 %	6	15 %	4
Dependence on medical treatment (4)	**9 %**	**1**	17 %	3
Energy (10)	6 %	4	11 %	7
Mobility (15)	5 %	5	14 %	5
Sleep (16)	2 %	7	12 %	6
Activities of daily living (17)	7 %	2	19 %	2
Work capacity (18)	7 %	2	**23 %**	**1**
**Psychological (item no.)**				
Enjoyment of life (5)	0 %	6	9 %	6
Personal belief (6)	1 %	5	**37 %**	**1**
Concentration (7)	**2 %**	**1**	17 %	5
Bodily image (11)	2 %	2	34 %	2
Self-esteem (19)	1 %	4	33 %	3
Negative feeling (26)	2 %	2	26 %	4
**Social Relationships (item no.)**				
Personal relationships (20)	0 %	3	26 %	2
Sexual activity (21)	**5 %**	**1**	**28 %**	**1**
Support from friends (22)	2 %	2	13 %	3
**Environment (item no.)**				
Security (8)	3 %	6	**22 %**	**1**
Physical environment (9)	3 %	6	17 %	2
Financial security (12)	9 %	2	3 %	8
Information availability (13)	6 %	4	7 %	4
Leisure activity (14)	**10 %**	**1**	7 %	4
Living conditions (23)	8 %	3	8 %	3
Health care accessibility (24)	1 %	8	6 %	6
Transport (25)	4 %	5	5 %	7
*Enough food (27)*	*2 %*		*12 %*	
*Extra item added to Chichewa WHOQoL-BREF*				

### Floor and ceiling effects

The proportion of respondents reporting the lowest (floor) and highest (ceiling) scores of each facet indicates which facets have the biggest effect on the overall measures of quality of life: the domain scores (Table 2). Table 2 also ranks the floor and ceiling effects within each domain to allow better visualization of which items affect their domain scores the most.

### Domain scores

The Environmental domain has the lowest score, followed by the Physical domain, the Social domain and the Psychological domain (Table 3; constituent facets of domain are in Table 2). The distributions of the Psychological, Social Relationships and Overall quality of life domain scores are more skewed towards 100 than those of the other domains. The added question regarding whether you have enough food to eat (item 27) was not included in the calculation of the Environment domain score so to facilitate cross-country comparisons with this study.

**Table 3:** Domain score distribution statistics

***Quality of Life Domain***	**min**	**max**	**mean**	**std. dev.**	**skewness**
Physical (PHYS)	3.6	100	60.3	19.59	−0.44
Psychological (PSYCH)	16.7	100	73.3	14.89	−0.89
Social Relationships (SOCIAL)	8.3	100	70.6	17.97	−0.66
Environmental (ENVIR)	9.4	93.8	57.6	16.82	−0.46
Overall (q1)	1	5	3.6	0.99	−0.73
Health (q2)	1	5	3.4	0.97	−0.25

### Internal consistency

Cronbach’s Alpha scores assess the internal consistency of each domain score based on the correlations between all responses to each of the questions of which the domains are comprised. Alpha scores greater than, or equal too, 0.7 are considered to denote adequate internal consistency. Using this criterion, all domains were found to have adequate internal consistency (see Table 4). Studies in other countries have noted problems with the internal consistency of the Social Relationships domain reporting Alpha scores as low as 0.55 [3-5]. This may be partly due to the fact that it is made up of only 3 items. Although this domain had the lowest Alpha score in our dataset (0.694), it was not significantly different to the threshold value of 0.7.

**Table 4:** **Correlation matrix of items and domains of the WHOQOL-BREF**^**a**^

	**Physical**	**Psychological**	**Social**	**Environment**	**Cronbach’s alpha**
**Physical (item no.)**					0.823
Physical Pain (3)	**0.67**	0.17	0.08	0.17	
Dependence on medical treatment (4)	**0.68**	0.25	0.09	0.23	
Energy (10)	**0.60**	0.27	0.06	0.18	
Mobility (15)	**0.72**	0.44	0.30	0.54	
Sleep (16)	**0.63**	0.56	0.46	0.57	
Activities of daily living (17)	**0.75**	0.31	0.17	0.32	
Work capacity (18)	**0.77**	0.31	0.20	0.27	
**Psychological (item no.)**					0.783
Enjoyment of life (5)	0.30	**0.72**	0.38	0.52	
Personal belief (6)	0.20	**0.74**	0.30	0.36	
Concentration (7)	0.28	**0.64**	0.33	0.44	
Bodily image (11)	0.36	**0.71**	0.29	0.39	
Self-esteem (19)	0.43	**0.73**	0.43	0.54	
Negative feeling (26)	0.36	**0.63**	0.43	0.53	
**Social Relationships (item no.)**					0.694
Personal relationships (20)	0.23	0.50	**0.80**	0.50	
Sexual activity (21)	0.42	0.48	**0.79**	0.37	
Support from friends (22)	0.09	0.30	**0.79**	0.43	
**Environment (item no.)**					0.828
Security (8)	0.38	0.50	0.30	**0.66**	
Physical environment (9)	0.20	0.53	0.32	**0.67**	
Financial secuity (12)	0.32	0.53	0.35	**0.71**	
Information availability (13)	0.40	0.51	0.36	**0.75**	
Leisure activity (14)	0.43	0.49	0.37	**0.70**	
Living conditions (23)	0.18	0.33	0.47	**0.63**	
Health care accessibility (24)	0.21	0.28	0.42	**0.60**	
Transport (25)	0.33	0.42	0.36	**0.68**	
*Enough food (27)*	*0.29*	*0.53*	*0.30*	***0.61***	
					

^a^Correlation of > 0.45 was considered satisfactory.

The validity of the domain structure was also assessed by calculating Pearson’s Correlation Coefficients for the relationship between each of the facet (question/item) scores and each of the four domain scores. All 27 items were found to be most correlated to the domain to which they are assigned, with all correlations greater than, or equal too, 0.60. This value, is significantly higher than the 0.40 [5] or 0.45 [11] threshold for acceptability given in other studies (Table 4).

### Associations with socio-demographic variables

Previous studies have shown WHOQOL-BREF domain scores to be associated with age (e.g. [14,15]) education (e.g. [15,16]), marital status (e.g. [15]) and gender (e.g. [14-16]) to differing extents in different populations [17]. Associations of these variables with each of the domain scores were assessed by univariate regression analysis (Table 5a). Notable associations include Physical domain, Psychological domain and Health domain scores deteriorating with age; all domain scores except Social Relationships being higher in those with tertiary education; the Physical, Psychological and Environmental domains being higher in those who are single (compared to married) and those living as married (although n = 6 for this group) who additionally have higher Health domain scores (Table 5a).

**Table 5:** Regressions of respondent characteristic variables including states assessing discriminant validity on quality of life domains

**a) Univariate Regressions**	**Physical domain (0–100 scale)**				**Psychological domain (0–100 scale)**				**Social domain (0–100 scale)**				**Environmental domain (0–100)**				**Overall domain (q1; 1–5 scale)**				**Health domain (q2; 1–5 scale)**			
(separate regr. for each variable)			95%CI				95%CI				95%CI				95%CI				95%CI				95%CI	
***Independent variable***	Coef.	p > |t|	lower	upper	Coef.	p > |t|	lower	upper	Coef.	p > |t|	lower	upper	Coef.	p > |t|	lower	upper	Coef.	p > |t|	lower	upper	Coef.	p > |t|	lower	upper
*Category: Reference = Sick(KCH)*																								
Healthy (KCH)	1.2	0.763	−6.7	−9.1	***−6.3***	***0.038***	***−12.4***	***−0.3***	2.1	0.568	−9.4	5.2	−4.9	0.157	−11.7	1.9	−0.33	0.105	−0.73	0.07	***0.47***	***0.018***	***0.08***	***0.86***
Healthy (village)	***9.0***	***0.032***	***0.8***	***17.2***	1.2	0.701	−5.0	−7.4	3.1	0.425	−4.5	10.6	−3.2	0.374	−10.3	3.9	−0.22	0.302	−0.63	0.20	0.29	0.160	−0.12	0.69
*Gender: Reference = male*																								
female	−2.7	0.237	−7.2	1.8	−1.9	0.270	−5.3	1.5	2.5	0.231	−1.6	6.6	0.7	0.720	−3.2	4.6	0.03	0.781	−0.20	0.26	0.11	0.328	−0.11	0.33
*Education: Reference = None*																								
Primary	3.4	0.467	−5.9	12.8	0.8	0.831	−6.4	8.0	1.8	0.694	−7.2	10.9	−1.0	0.817	−9.3	7.3	0.19	0.450	−0.30	0.67	0.33	0.179	−0.15	0.82
Secondary	**12.6**	**0.007**	**3.5**	**21.7**	7.2	0.440	0.2	14.2	1.8	0.681	−7.0	10.7	3.8	0.357	−4.3	11.9	0.34	0.159	−0.13	0.82	0.42	0.081	−0.05	0.90
Tertiary	21.6*	2E-05*	11.7*	31.6*	13.4*	6E-04*	5.8*	21.1*	6.6	0.177	−3.0	16.3	***9.9***	***0.029***	***1.0***	***18.7***	**0.82**	**0.002**	**0.30**	**1.34**	***0.59***	***0.027***	***0.07***	***1.10***
*Marital Status: Reference = Married*																								
Single	***6.8***	***0.010***	***1.6***	***12.0***	7.3*	4E-04*	3.3*	11.3*	−0.4	0.885	−5.4	4.6	**6.9**	**0.003**	**2.3**	**11.6**	0.27	0.057	−0.01	0.54	0.12	0.399	−0.15	0.38
Living as Married	**20.1**	**0.003**	**6.8**	**33.4**	**15.2**	**0.004**	**5.0**	**25.5**	11.6	0.075	−1.2	24.3	**15.9**	**0.008**	**4.1**	**27.6**	0.30	0.399	−0.40	1.00	***0.73***	***0.036***	***0.05***	***1.42***
Separated	−16.0	0.432	−16.0	6.8	−4.7	0.293	−13.5	4.1	−6.7	0.230	−17.7	4.3	0.4	0.943	−9.7	10.5	−0.30	0.325	−0.90	0.30	−0.48	0.107	−1.07	0.10
Divorced	−1.0	0.858	−11.5	9.6	1.3	0.750	−6.8	9.5	2.4	0.648	−7.8	12.5	3.3	0.488	−6.0	12.6	0.12	0.678	−0.44	0.67	0.07	0.802	−0.47	0.61
Widowed	−15.0*	1E-04*	−22.6*	−7.4*	−3.8	0.206	−9.6	2.1	−2.2	0.553	−9.5	5.1	−1.2	0.725	−7.9	5.5	−0.06	0.783	−0.46	0.34	−0.28	0.157	−0.67	0.11
*Ill: Reference category = not ill*	−4.9	0.103	−10.9	1.0	2.7	0.238	−1.8	7.2	−0.4	0.893	−5.8	5.1	4.1	0.116	−1.0	9.2	0.28	0.071	−0.02	0.58	***−0.38***	***0.010***	***−0.68***	***−0.09***
*Age (years)*	−0.44*	4E-08*	−0.59*	−0.29*	**−0.17**	**0.007**	**−0.29**	**−0.46**	−0.02	0.745	−0.17	0.12	−0.129	0.064	−0.266	0.008	−0.003	0.460	−0.011	0.005	−0.017*	2E-05*	−0.025*	−0.009*
*Age*^*2*^	**−0.0053**	**5E-09**	**−0.0070**	**−0.0036**	***−0.0017***	***0.015***	***−0.0031***	***−0.0033***	−8E-05	0.927	−0.002	0.002	−0.001	0.092	−0.003	0.0002	−1E-05	0.803	0.000	−8E-05	−0.0002*	3E-05*	−0.0003*	−0.0001*
*q1 (overall QoL, per 1 on 1–5 scale)*	6.4*	6E-09*	4.3*	8.5*	8.7*	0*	7.3*	10.1*	6.8*	0*	4.9*	8.7*	11.1*	0*	9.6*	12.6*					0.41*	0*	0.31*	0.51*
*q2 (overall health, per 1 on 1–5 scale)*	7.2*	1E-10*	5.0*	9.3*	4.2*	1E-06*	2.6*	5.9*	4.1*	1E-04*	2.0*	6.1*	4.4*	7E-06*	2.5*	6.3*	0.43*	0*	0.32*	0.53*				
*q26 (Depression, per 1 on 1–5 scale)*	8.0*	0*	5.8*	10.3*	10.5*	0*	9.1*	11.9*	8.7*	**0**	6.7*	10.7*	10.0*	0*	8.3*	11.8*	0.47*	0*	0.37*	0.58*	0.27*	6E-06*	0.16*	0.39*
*Disability weight of illness (0–1 scale)*	**−23.3**	**0.005**	**−39.4**	**−7.1**	3.7	0.536	−8.1	15.6	4.0	0.564	−9.7	17.7	8.7	0.18	−4.0	21.5	0.4	0.304	−0.4	1.2	***−0.8***	***0.042***	***−1.5***	***0.0***
**b) Multivariate Restricted models**^**a**^																								
*Category: Reference = Sick(KCH)*																								
Healthy (KCH)					***−6.0***	***0.043***	***−11.7***	***0.2***																
Healthy (village)					2.0	0.512	−3.9	7.9	no variables significantly associated with social relationships domain															
*Gender: Reference = male*																								
female																								
*Education: Reference = None*																								
Primary	0.2	0.966	−9.0	9.4	0.8	0.819	−6.3	7.9									0.33	0.182	−0.16	0.82				
Secondary	5.0	0.297	−4.4	14.4	4.9	0.171	−2.1	12.0									***0.53***	***0.038***	***0.03***	***1.04***				
Tertiary	**14.6**	**0.005**	**4.4**	**24.8**	**11.2**	**0.005**	**3.4**	**18.9**									1.03*	2E-04*	0.48*	1.57*				
*Marital Status: Reference = Married*																								
Single	0.9	0.746	−4.5	6.3	***4.8***	***0.022***	***0.7***	***8.9***					**6.9**	**0.003**	**2.3**	**11.6**								
Living as Married	***17.0***	***0.010***	***4.0***	***29.9***	**14.3**	**0.006**	**4.1**	**24.4**					**15.9**	**0.008**	**4.1**	**27.6**								
Separated	−5.6	0.313	−16.5	5.3	−5.5	0.209	−14.0	3.1					0.4	0.943	−9.7	10.5								
Divorced	−2.5	0.629	−12.7	7.7	−0.7	0.855	−8.7	7.2					3.3	0.488	−6.0	12.6								
Widowed	−8.0	0.053	−16.1	0.1	−2.5	0.399	−8.3	3.3					−1.2	0.725	−7.9	5.5								
Ill: Reference category = not ill																								
*Age (years)*																	**−0.068**	**0.002**	**−0.111**	**−0.025**	−0.017*	2E-05*	−0.025*	−0.009*
*Age*^*2*^	***−0.0027***	***0.012***	***−0.0049***	***−0.0006***													**0.001**	**0.001**	**0.000**	**0.001**				
*R*^*2*^			0.196				0.150				0.018				0.049				0.088				0.057	
**c) Variables added to multivariate restricted models**^**b**^																								
*q1 (overall QoL, per 1 on 1–5 scale)*	3.6*	0.001*	1.4*	5.8*	7.7*	0*	6.2*	9.2*	6.2*	1E-08*	4.1*	8.3*	11.1*	**0**	9.5*	12.7*					0.4*	0*	0.3*	0.5*
*q2 (overall health, per 1 on 1–5 scale)*	4.0*	5E-04*	1.8*	6.2*	0.3	0.660	−1.2	1.9	1.4	0.190	−0.7	3.5	−0.7	0.409	−2.3	0.9	0.43*	0*	0.33*	0.53*				
*q26 (Depression, per 1 on 1–5 scale)*	6.1*	9E-08*	3.9*	8.3*	9.6*	0*	8.2*	11.1*	as univariate above				9.8*	0*	7.9*	11.5*	0.45*	0*	0.34*	0.56*	0.24*	5E-05*	0.12*	0.35*
*Disability weight of illness (0–1 scale)*	**−17.5**	**0.026**	**−33.0**	**−2.1**	4.4	0.479	−7.8	16.6	as univariate above				9.4	0.142	−3.2	22.1	0.26	0.513	−0.51	1.02	−0.56	0.134	−1.30	0.17

***p ****< ****0.05 *p **< 0.01 *p < **0.001 **. E=exponent (base 10) e.g. 2E-06 = 2 x 10^-6^.

^a^ Restricted model after starting with all socio-demographic variables and removing those p > 0.1 significant.

^b^ q1 and q2 added together; other coefficients not shown (but generally not qualitatively different from section b above after section c variables added).

Subsequent multivariate analyses (Table 5b) show that many of the univariate associations are not significant when controlling for other variables. Physical domain scores are higher in those with tertiary education and for those living as married (compared to married) and lower in those who are older, especially at older ages (age^2^ is negative and significant). Psychological domain scores are lower in healthy respondents attending patients at KCH (as also shown in numerous previous studies [18]) higher in tertiary educated respondents and those single or living as married. Social domain scores were not significantly associated with any of the variables assessed. Environmental domain scores are higher in those single or living as married. Overall domain scores are slightly higher in those with secondary and tertiary education. Health domain scores are lower in older respondents (Table 5b).

The R^2^ values for all of the regression models were fairly low meaning that the variation in each domain score was not well explained by the socio-demographic variables included in the model alone. This is perhaps as expected, considering that many more factors are likely to influence an individuals quality of life than gender, age, education and marital status.

### Discriminant validity

This section examines associations between the four main domain scores and overall quality of life (q1), health (q2), depression (q26), and disability weights associated with respondents specific health problems (see methods). Associations were determined by univariate regression and by addition of the variables to restricted multivariate models as described in the methods.

Overall quality of life is positively associated with all four main domain scores and the Health domain score (increase of 6–11 points on the 0–100 scale of the four main domains and 0.4 on the 1–5 scale of Health domain, per 1 increase on 1–5 scale of the Overall domain—univariate regressions: Table 5a; multivariate regressions show slightly lower increases: Table 5c).

The Health domain is also positively associated with all four domains and the Overall quality of life domain (increase of 4–7 points on the 0–100 scale of the four main domains and 0.4 on the 1–5 scale of the Overall domain, per 1 increase on the 1–5 scale of the Health domain—univariate regressions: Table 5a). However, after controlling for the remaining variables from the restricted multivariate regressions (Table 5b) and for Overall quality of life, the associations with the Psychological, Social, and Environmental domains disappear (Table 5c).

Depression (q26) was found to be significantly associated with all six domains (Physical, Psychological, Social and Environmental domains scores (0–100 scales) were between 8.0 and 10.5 points higher per 1 increase on the 1–5 scale of Never depressed (1), seldom depressed (2), quite often depressed (3), very often depressed (4) and always depressed (5); and 0.47 and 0.27 points higher on Overall QoL and Health domains respectively per 1 increase on the 1–5 scale -univariate regressions in Table 5a; similar for multivariate regressions in Table 5c).

Respondents with current health conditions with higher (worse) disability weights were found to have significantly lower Physical domain scores only (−2.33 on the 0–100 scale per 0.1 increase in disability; 95%CI: −3.94, −0.71; p = 0.005) and overall Health domain scores (−0.08 on the 1–5 scale per 0.1 increase in disability; 95%CI: −0.15, −0.00; p = 0.042; Table 5a univariate regressions; slightly lower decreases for multivariate analyses: Table 5c).

## Discussion

This study has examined the psychometric properties of a newly translated Chichewa version of the WHOQOL-BREF among healthy adults and adults with a range of different health conditions from the general population of Malawi. It provides information on the acceptability, comprehensibility and validity of the Chichewa WHOQOL-BREF.

### Acceptability and comprehensibility

The high response rate of 97 % and the fact that nearly all-300 respondents answered all questions except q21 on sexual activity indicates high general acceptability and comprehensibility of the questionnaire. As with other studies (e.g. [5]) q21 was often refused, in this case by 36 % of respondents, due to it’s sensitive nature. Comments by those who refused Q21 included: *“Question 21 is a taboo”* and “*Question 21 ought to be selective depending on somebody’s age”*.

Regarding comprehensibility, there were only a handful of comments by those who did not understand specific questions: “*Questions 17* [activities of daily living] *and 18* [work capacity] *are difficult to understand*”; regarding question 12 [financial security]: “*There is no way a person can be satisfied with money. The question might as well be dropped”*; “*On question 3* [physical pain] *it depends on the severity of the sickness”*; regarding question 26: “*consider splitting… …some may be unhappy but optimistic. One answer cannot stand for all those feelings”*; and, *“I wonder whether some questions relate to good quality of life, for instance question 21”*. Respondents were invited to provide these comments at the end of the questionnaire.

### Validity

The internal consistency and structure of the four domains of the Chichewa WHOQOL-BREF was validated by analyses showing that each item was most correlated to the domain assigned to it by the original authors of the WHOQOL-BREF.

Evidence for the ability of the Chichewa WHOQOL-BREF to discriminate between known groups was also found. All four domain scores differed significantly and by large amounts in the expected direction depending on how depressed the respondent was, the disability weight of their current health condition, and their overall quality of life.

The findings regarding disability weights also fulfill the main WHO-mandated goal of this pilot study [9] by illustrating the discriminative nature of the Chichewa version of the WHOQOL-BREF with regard to determining the effect of different states of health (often the target of a specific intervention being evaluated) on quality of life. As expected, respondents with health states with higher disability weights were found to have lower physical quality of life scores.

The association between low quality of life and high disability weights, also provides evidence of convergent validity in that the WHOQOL-BREF scores were associated with the scores, given by people in similar health states, that were originally used to construct the disability weights [19]. To our knowledge, alternative instruments for assessing quality of life such as the EQ-5D, or the SF-36 which is commonly used for comparison with the WHOQOL-BREF [5,20] are not available in Malawi.

### Reliability

Test-retest reliability was not examined in this study. Despite the fact that others have attempted to assess this (e.g. [5]), we considered that running the survey as a patient interview precluded test-retest reliability assessment, as being out of hospital, even one day after the initial interview, might change the results obtained.

### Other findings

Analysis of how the domain scores varied depending on socio-demographic characteristics yielded some interesting results. Older respondents were found to have lower Physical and Psychological quality of life—although when controlling for gender, education and marital status older age was only associated with lower Physical domain scores. A higher level of educational attainment was found to be associated with higher quality of life in all domains except for Social Relationships and these associations (except for that with Environment domain scores) held constant after controlling for the other socio-demographic variables. Respondents living as married or single were found to have higher quality of life in the Physical, Psychological and Social domains, and those who were widowed lower Physical quality of life.

The positive relationships between education (especially tertiary) and domain scores (Physical, Psychological and Overall after controlling for other significant variables) are also interesting. It’s possible that these relationships are mediated by better education leading to higher earnings, which lead to better health and higher quality of life. Recent studies in poor rural communities in older adults also show lower self-reported health and quality of life in those with lower education and lower socio-economic status [21-25]. In this study it is uncertain how self-reported quality of life differed compared to actual quality of life (although possibilities are discussed below) and whether this is dependent on education or other variables.

It is also interesting to note that the correlation between age and education is highly statistically significant (p = 1.4 × 10^−12^) and negative (Pearson’s correlation = −0.4; data not shown), meaning that older respondents had attained a lower level of education as may perhaps be expected given recent advances in access to education in Malawi.

### Limitations

There was some overlap between the sample categories in the sense that: all 26 healthy people interviewed in KCH were guardians of patients and as such may have been affected by the sickness of their friends/relatives; eight of the 259 ‘sick’ respondents were caring for sick children rather than sick themselves although they perceived themselves to be ill; and six respondents who answered ‘*No*’ to the initial ‘*Are you currently ill?*’ question still defined themselves as having specific (perhaps asymptomatic) health problems when asked in the next question (Additional file 2). These anomalies are unlikely to have affected the results significantly.

Disability weights were not available for all health conditions specified by respondents; and some of the conditions were difficult to match with those listed due to insufficient information provided by the respondent. Future studies would benefit from collecting more information to better match the respondent’s condition to those on the global burden of disease list.

The multivariate disability weight and depression analyses were done by adding the variable in question to the already arrived at restricted socio-demographic model, i.e. they assume that a positive value of the added variable in question does not predate the socio-demographic variables. Whilst this should be valid in most cases it is invalid for cases, which remain unknown, where the disability/depression has been very long term and pre-dates education and marital status for example, in which case the disability/depression variables should be added to the regression at the beginning with the other variables.

Despite the validity checks it’s possible that there is a bias in the answer to some questions and particularly those of the Psychological domain (which has a higher mean score and is skewed towards 100) due to anecdotal evidence of an ‘it is well with my soul’ syndrome in Malawians. It is hypothesized that for all questions that are direct to an individual’s well being, a Malawian will prefer to respond that they are ok, when they are not ok. In Malawi, as in other countries, the usual greeting is *‘muli bwanji?* (how are you?) and the responses are always *‘ndli bwino khaya inu’* (I’m fine what about you?) even if they are sick, hungry, without money or frustrated. Anecdotal evidence suggests Malawian’s generally only say things are not ok after further questions. For questions concerning far away issues (e.g. money, living places, transport—Environment domain) the scoring may be poorer as anecdotal evidence suggests Malawians are generally more honest with distant things than things within themselves. These potential biases can’t be ruled out by the quantitative validation methodology employed in this study.

Finally, because the survey was not conducted among a representative sample of the general population, it is not possible to conclude that the domain and facet scores from this sample reflect the quality of life of the general population of Malawi.

## Conclusions

This study provides evidence that the newly translated Chichewa version of the WHOQOL-BREF is valid for future use within Malawi. It is hoped that subsequent evaluations of health states, health interventions and wider interventions in the public sphere will benefit from its use.

## Abbreviations

KCH: Kamuzu Central Hospital (Lilongwe Malawi); NHSRC: National Health Sciences Research Council (Malawi ethics board); q/Q: Question (on the WHOQOL-BREF); PACHI: Parent and Child Health Initiative (Malawi); QoL: Quality of Life; UCL: University College London; UK: United Kingdom of Great Britain and Northern Ireland; WHO: World Health Organisation; WHOQOL-BREF: WHO Quality of Life questionnaire—short version.

## Competing interests

We declare that we have no conflicts of interest.

## Authors’ contributions

TC and JSW conceived the study, which was then designed by TC, GM and JSW. TC and GM planned and oversaw the data collection and data entry. TC carried out all analyses with input from JSW. TC wrote the first draft of the paper and all authors contributed to subsequent drafts and agreed to the final manuscript.

## Supplementary Material

Additional file 1 Chichewa WHOQOL-BREF. Click here for file

Additional file 2 Disability weights of self-reported health conditions of respondents. Click here for file

Additional file 3 **Table S1.** Frequency of each response to each of the questions of the WHOQOL-BREF. Click here for file
