# Supplementary material for: Development, reliability and validity of the Chichewa WHOQOL-BREF in adults in lilongwe, Malawi
Source: BMC Res Notes. 2012 Jul 3;5:346. doi: 10.1186/1756-0500-5-346 (PMC3483688; doi:10.1186/1756-0500-5-346)
Supplement: Additional file 1 — Chichewa WHOQOL-BREF. [file 1756-0500-5-346-S1.pdf]

The WHOQoL-BREF I.D. number | | | | |

Tikudziweni

Musanayambe, tikadakonda mutayankha mafunso pang'ono okhudzana ndi inu: pozunguliza yankho lolondola kapena polemba yankholo m'mizere yotsatilayi.

Kodi ndinu amuna kapena akazi? Amuna Akazi

Kodi tsiku lanu lakubadwa ndiliti? _______/______/________
Tsiku Mwezi Chaka

Kodi munafika pati ndi maphunziro anu?
- sindinapitepo ku sukulu
- pulayimale sukulu
- sekondale sukulu
- maphunziro opitilira sekondale sukulu

Kodi muli pabanja?
- sindinakwatirepo
- okwatira
- timakhala limodzi koma tilibe setifiketi ya kutchalitchi kapena kwa DC
- tinasiyana koma banja silinathe
- banja linatha
- namfedwa

Kodi panopa mukudwala?
- eya
- ayi

Ngati muli ndi vuto la umoyo mukuganiza kuti ndichani? ____

Malangizo

Mafunso otsatilawa akufuna kudziwa za zomwe mukumvera zokhudzana ndi kupambana kwa moyo wanu, umoyo kapena zinthu zina zokhudza moyo wanu. **Chonde yankhani mafunso** onse. Ngati mukukayikira yankho limene mukufuna mupereke, chonde sankhani lomwe likuwoneka ngati lokhonza. Nthawi zambiri yankholi limakhala lomwe munaliganizila poyamba.

Chonde kumbukirani mulingo omwe mumadziyika, ziyembekezo zanu, zomwe zimakusanagalatsani, ndi nkhawa zanu. Tikufunsani kuti muganizire za moyo wanu **m'sabata ziwiri zapitazi**. Mwachitsanzo, poganizira masabata awiri apitawa, mukhonza kufunsidwa:

	Kodi mumalandira chinthandizo choyenera chimene mumafuna kuchokera kwa anthu ena?	Ayi<br>1	Pang'ono<br>2	Pakatikati<br>3	Kwambiri<br>4	Chonse<br>5

Muzungulize nambala yomwe ikulongosola bwino kuchuluka kwa chithandizo chomwe munalandira kuchokera kwa anthu ena m'sabata ziwiri zapitazi. Choncho munakazungi nambala 4 ngati munalandira chithandizo kwambiri kuchokera kwa anzanu ena motere.

	Kodi mumalandira chinthandizo choyenerera chimene mumafuna kuchokera kwa anthu ena?	Ayi<br><br>1	Pang'ono<br><br>2	Pakatikati<br><br>3	Kwambiri<br><br>4	Chonse<br><br>5

Munakazunguliza nambala 1 ngati simunalandire chithandizo chilichonse chomwe munafuna kuchokera kwa anthu ena m'sabata ziwiri zapitazi. Chonde werengani funso lililonse, ganizilani za malingaliro anu ndipo zungulizani namabala pa mulingo omwe yankho lolondola kwa inuyo layikidwa pa funso lililonse.

THE WHOQOL-BREF

		Sulibwino kwambiri	Sulibwino	Uli pakatikati	Ulibwino	Ulibwino kwambiri
1 (G1)	Mukaziona, moyo wanu ndiwapambana bwanji?	1	2	3	4	5

		Osakhutitsidwa kwambiri	Osakhutitsidwa	Pakatikati	Okhutitsidwa	Okhutitsdwa kwambiri
2 (G4)	Kodi ndinu okhutira bwanji ndi umoyo wanu?	1	2	3	4	5

Mafunso otsatilawa akufunsa za mulingo wa zina zomwe mwakumana nazo m'sabata ziwiri zapitazi.

		Palibe/ayi	Pang'ono	Pakatikati	Kwambiri	Kwambiri zedi
3 (F1.4)	Mukuona ngati kuwawa kwa mthupi kwanu kwakulepheretsani bwanji kuchita zomwe mumafuna kuchita?	1	2	3	4	5
4 (11.3)	Kodi mumafuna chithandizo cha chipatala chochuluka bwanji kuti muchite zofunika kuchita tsiku ndi tsiku?	1	2	3	4	5
5 (F4.1)	Kodi mumasangalala kwambiri bwanji ndi moyo?	1	2	3	4	5
6 (F24.2)	Kodi mukuganiza kuti moyo wanu ndi watanthauzo motani?	1	2	3	4	5

		Ayi/Palibe	Pang'ono	Pakatikati	Kwambiri	Kwambiri zedi
7 (F5.3)	M'makhala ndi chidwi (chomvetsera) choyenera bwanji pakuchita zinthu?	1	2	3	4	5
8	Kodi mumaona kuti ndinu	1	2	3	4	5

		Ayi/Palibe	Pang'ono	Pakatikati	Kwambiri	Kwambiri zedi
(F16.1)	otetezedwa bwanji pa moyo wanu wa tsiku ndi tsiku?					
9 (F22.1)	Kodi malo amene mumapezeka kapena kukhala kawirikawiri ndi abwino bwanji ku umoyo wanu?	1	2	3	4	5

Mafunso otsatilawa akufuna kudziwa kuti mwakwanitsa bwanji komanso munatha bwanji kuchita zina ndi zina m'sabata ziwiri zapitazi.

		Tilibe/Ayi	Pang'ono	Pakatikati	Kwambiri	Kwambiri zedi
10 (F2.1)	Kodi muli ndi mphamvu zokwanira zochitila zinthu tsiku ndi tsiku?	1	2	3	4	5
11 (F7.1)	Kodi mutha kuvomereza m'mene maonekedwe anu alili?	1	2	3	4	5
12 F(18.1)	Kodi mumakhala ndi ndalama zokwanira kuti mukwanitse zofunikira?	1	2	3	4	5
13 (F20.1)	Kodi muli ndi mwayi otani wotha kupeza zinthu zokuphunzitsani zimene mumafuna pa moyo wanu	1	2	3	4	5
14 (F21.1)	Kodi muli ndi mwayi wotani wochita zinthu za nsangulutso?	1	2	3	4	5
**27**	Kodi mumakhala ndi chakudya chokwanira kudyetsa banja lanu?	1	2	3	4	5

		Ndikovuta kwambiri	ndikovuta	pakatikati	Ndikophweka	Ndikophweka kwambiri
15 (F9.1)	Kodi ndikophweka bwanji kwa inuyo kutha kuyendayenda?	1	2	3	4	5

Mafunso otsatiliwa akufuna kudziwa m'mene mwamvera ubwino kapena m'mene mwakhutitsidwira ndizochitika zosiyanasiyana za moyo wanu m'sabata ziwiri zapitazi

		Osakhutitsi-dwa kwambiri	Osakhutit-sidwa	Pakatikati	Okhutitsidwa	Okhutitsidwa kwambiri
16 (F3.3)	Kodi ndinu okhutitsidwa bwanji ndi tulo timene mumapeza mukagona?	1	2	3	4	5
17 F(10.3)	Ndinu okhutitsidwa bwanji ndi m'mene mungakwanilitsire kugwira ntchito zanu za tsiku ndi tsiku?	1	2	3	4	5
18 (F12.4)	Kodi mumakhutitsidwa bwanji ndi m'mene mumangakwanilitsire kugwira ntchito?	1	2	3	4	5

		Osakhutitsi -dwa kwambiri	Osakhutit -sidwa	Pakatikati	Okhutitsidwa	Okhutitsidwa kwambiri
19 (F6.3)	Kodi ndinu okhutitsidwa bwanji ndinu mwini?	1	2	3	4	5
20 (F13.3)	Ndinu okhutitsidwa bwanji ndi m'mene ubale wanu ulili ndi anthu ena?	1	2	3	4	5
21 (F15.3)	Ndinu okhutitsidwa bwanji ndi moyo wanu ogonana ndi achikondi anu?	1	2	3	4	5
22 (F14.4)	Ndinu okhutitsidwa bwanji ndi chithandizo chomwe mumalandira kuchokera kwa anzanu?	1	2	3	4	5
23 (F17.3)	Ndinu okhutitsidwa bwanji ndi m'mene malo anu mumakhala alili?	1	2	3	4	5
24 (F19.3)	Muli okhutira bwanji ndi kupezeka kwa chithandizo cha za umoyo?	1	2	3	4	5
25 (F23.3)	Muli okhutitsidwa bwanji ndi zokhudza ndi mayendedwe (tharasipoti)?	1	2	3	4	5

Funso lotsatilalri likukhudzana ndi m'mene mwamvera kapena kudutsana ndi zinthu zina kawirikawiri bwanji m'sabata ziwiri zapitazi.

		Sizinachiti-kepo	Mwapatali-patali	Kawirikawiri	Kawirikawiri kwambiri	Nthawi zonse
26 (F8.1)	Kodi ndi kawirikawiri bwanji pomwe mumakhala osakondwa monga kukhala a chisoni, otaya mtima, odandauladandaula, kapena okhumudwa?	1	2	3	4	5

Kodi alipo anakuthandizani kuyankha mafunsowa?

____

Zinakutengerani nthawi yayitali bwanji kuti mumalize kuyankha mafunsowa?

____

Kodi muli ndi ndemanga iliyonse yokhudzana ndi mafunsowa?

____

____

____

Zikomo kwambiri pakutenga mbali kwanu
