# Supplementary material for: Development, reliability and validity of the Chichewa WHOQOL-BREF in adults in lilongwe, Malawi
Source: BMC Res Notes. 2012 Jul 3;5:346. doi: 10.1186/1756-0500-5-346 (PMC3483688; doi:10.1186/1756-0500-5-346)
Supplement: Additional file 2 — Disability weights of self-reported health conditions of respondents. [file 1756-0500-5-346-S2.pdf]

Web Appendix 2. Disability weights of self-reported health conditions of respondents

Health problem				Number of cases		
Category	Specific problem	Disability Weight	Notes	WHO pilot - Healthy (KCH)	WHO pilot - Healthy (village)	WHO pilot - Sick (KCH)
**Health problem Not Stated**		NA		-	-	31
**Anaemia / weakness**	anaemia	0.090	Severe iron deficiency anemia (assuming this as patient is in hospital); Maternal Haemorrhage severe anemia is 0.093; Hookworm anemia is 0.024; Malaria anemia is 0.012	-	-	4
	dizziness	Not listed		-	-	3
	general body weakness	Not listed		-	-	2
	low blood pressure	Not listed		-	-	1
	loss of appetite	Not listed		-	-	4
**Cancer**	brain tumor	0.750	from disability weights PDF: Note: For all cancer sites, the disability weight is 0.75 for the metastasis stage, and 0.81 in the terminal stage; assuming at least metastasis stage as patient is in hospital	-	-	1
	cancer	0.750	as above	-	-	1
	cervical cancer	0.750	as above	-	-	1
	tumor	0.750	as above	-	-	3
	tumor in the stomach	0.750	as above	1	-	1
**Child sick**	child burnt	Not listed		-	-	3
	child sick (born with lip problem and got operated)	Not listed		-	-	1
	child sick (eye problem)	Not listed		1	-	0
	child sick (hit with a stone)	Not listed		1	-	0
	child sick (scar not hardening)	Not listed		-	-	1
	child with a fracture	Not listed		-	-	1
	child with a fractured leg	Not listed		-	-	1
	child's head abnormal growth	Not listed		-	-	1
**Diabetes**	diabetes	0.215	ranges from 0.015 for a case of diabetes to 0.072 for diabetes neuropathy, 0.102 for diabetes amputation, 0.133 for diabetic foot, and 0.552 for diabetes Retinopathy (blindness). Given these cases are in hospital for diabetes they should probably be put as the avergae of all the severe forms: (0.072+0.102+0.133+0.552)/4=0.215	-	-	2
	sugar disease (diabetes)	0.215		-	-	2
**Fever / Malaria**	fever	0.191	Malaria episodes is 0.191, Neurological Sequelae is 0.471, Malaria anemia is 0.012. Given that the person is in hospital for fever we are assuming it is as bad as Malaria (aslo, dengue fever episodes are similar at 0.197)	-	-	5
	malaria	0.191		-	-	20
	sweating	Not listed		-	-	1
**Hypertension / Heart attack / Stroke**	heart attack	0.439	Acute Myocardial infarction	-	-	1
	high blood pressure	0.246	Hypertensive heart disease - cases	-	-	6
	hypertension	0.246	Hypertensive heart disease - cases	-	-	1
	stroke	0.920	First-ever stroke cases is 0.920; Long-term stroke survivors are 0.266. Given the person is in hospital we will take the first value (although it seems to high for the person to be interviewed!)	-	-	2
**Infection**	boil at the back	0.006	High-intensity infections for Hookworm, Ascariasis and Trichuriasis are all 0.006; Schistosomiasis infection is 0.005; others are 0.000	-	-	1
	boil on the breast	0.006	High-intensity infections for Hookworm, Ascariasis and Trichuriasis are all 0.006; Schistosomiasis infection is 0.005; others are 0.000	-	-	1
	cervical infection	0.067	Reproductive tract infection	-	-	1
	cold flu	0.191	Malaria episodes is 0.191, Neurological Sequelae is 0.471, Malaria anemia is 0.012. Given that the person is in hospital for fever we are assuming it is as bad as Malaria (aslo, dengue fever episodes are similar at 0.197); influenza is not specifically listed but from my experience it is about as bad as malaria!	-	-	2
	dental infection	Not listed		-	-	1
	diarrhoea	0.006	High-intensity infections for Hookworm, Ascariasis and Trichuriasis are all 0.006; Schistosomiasis infection is 0.005; others are 0.000	-	-	7
	ear infection	0.023	Otitis media - chronic infection	-	-	5
	eye infection	0.108	Injury to eyes	-	-	1
	eye problem	0.108	Injury to eyes	-	-	2
	eye problem (can't see)	0.108	Injury to eyes	-	-	1
	intestinal infection	0.006	High-intensity infections for Hookworm, Ascariasis and Trichuriasis are all 0.006; Schistosomiasis infection is 0.005; others are 0.000	-	-	1
	kidney infection	0.104	Schistosomiasis - Advanced renal disease	-	-	3
	measles	0.152	Measles - Episodes	-	-	1
	meningitis	0.615	Streptococcus pneumoniae - Episodes: 0.615; Haemophilus influenzae - Episodes: 0.616; Neisseria meningitidis - Episodes: 0.615	-	-	1
	pneumonia	0.279	Lower respiratory infections - Episodes: 0.279	-	-	1
	respiratory system infection	0.279	Lower respiratory infections - Episodes: 0.279 (Uper respiratory infections - Episodes: 0.000 (so unlikely to be in hospital)	-	-	1
	shingles	Not listed		-	-	1
	sore throat	0.070	Upper respiratory infections - Pharyngitis	-	-	1
	sores on the throat	0.070	Upper respiratory infections - Pharyngitis	-	-	3
	STI	0.067	Reproductive tract infection	1	-	3
	urinary bladder infection	0.067	Reproductive tract infection	-	-	1
**loss of memory**		Not listed		-	-	1

continued on next page

web appendix 2 continued:

Health problem		Disability Weight	Notes	Number of cases		
Category	Specific problem			WHO pilot - Healthy (KCH)	WHO pilot - Healthy (village)	WHO pilot - Sick (KCH)
**Pain / Physical injury**	abdominal pains	0.082	The only two categories of pain listed are: Low back pain=0.061 and Chronic Pelvic pain=0.122Also, Sprains are 0.064. Could assume all other pains are avearge of these three. Have done analyses with and without these assumptions	-	-	3
	anal pain	0.082	as abdominal pains above	-	-	1
	backache	0.061	Low back pain	-	-	2
	bleeding	0.108	Open wound	-	-	2
	body pains	0.082	as abdominal pains above	-	-	1
	chest pains	0.082	as abdominal pains above	-	-	3
	consistent body itching			-	-	1
	fractured arm	0.180	Fracture: Ulna or radius	-	-	2
	fractured leg	0.271	Patella, tibia or fibula	-	-	1
	general body pains	0.082	as abdominal pains above	-	-	4
	headache	0.082	as abdominal pains above	-	-	6
	headache, general body pains and dizziness	0.082	as abdominal pains above	-	-	1
	hernia	0.061	Herniation	1	-	3
	joint pains	0.082	as abdominal pains above	-	-	1
	kidney problem	0.104	Schistosomiasis - Advanced renal disease	-	-	1
	leg-ache	0.082	as abdominal pains above	-	-	2
	muscle cramps	0.082	as abdominal pains above	-	-	2
	neck stiffness	0.082	as abdominal pains above	-	-	1
	nose bleeding		This is not as bad as 'open wound' is it?	-	-	2
	pain after operation delivery	0.082	as abdominal pains above	-	-	1
	pain when passing out urine	0.122	Chronic pelivic pain (Chlamydia and Gonorrhea)	-	-	1
	stomach ache	0.082	as abdominal pains above	-	-	2
	stomach pains	0.082	as abdominal pains above	-	-	1
	throat sores and general body pains	0.070	Upper respiratory infections - Pharyngitis	-	-	1
	wound on thigh	0.108	Open wound	-	-	1
**Pregnancy and Childbirth**	after birth stomach complications	Not listed		-	-	1
	delivery by scizor	Not listed		-	-	1
	labour complications	Not listed		-	-	5
	operation (removal of uterus)	Not listed		-	-	1
	post delivery complications	Not listed		-	-	1
	pregnant	Not listed		-	-	1
**Respiratory Problems**	asthma	0.043	Asthma - cases	-	-	5
	bronchitis	0.279	Lower respiratory infections - Episodes: 0.279	-	-	1
	cough		Not sure whether this case is upper respiratory (weight=0.000) or Lower respiratory (weight=0.271)	-	-	6
	coughing		as above	-	-	1
	difficulties in breathing	0.279	Lower respiratory infections - Episodes: 0.279	-	-	2
	difficulty in breathing	0.279	Lower respiratory infections - Episodes: 0.279	-	-	1
	TB	0.271	Tuberculosis - cases	-	-	7
**Stomach complications / vomitting**	abdominal complications	Not listed		-	-	1
	consistent vomiting	Not listed		-	-	1
	eating disorders	Not listed		-	-	1
	failure to pass out stool	0.024	Ascariasis (Intestinal obstruction)	-	-	1
	intestinal complication	Not listed		-	-	1
	spleen disfunction	Not listed		-	-	1
	stomach complication	Not listed		-	-	1
	stomach complications	Not listed		-	-	5
	purging	Not listed		-	-	1
	ulcers	0.042	Peptic ulcer disease - Cases not treated with antibiotic	1	-	7
	vomiting	Not listed		-	-	3
**Swelling / inflammation**	Elephantiasis (swollen legs)	0.111	Lymphatic filariasis: Bancroftian lympoedema=0.106; Brugian lympoedema=0.116; since it is unclear which one it is and they are quite similar, I have used the mid-point 0.111	-	-	1
	skin disease	0.056	Skin diseases - cases	-	-	4
	swollen belly	Not listed		-	-	2
	swollen leg	0.111	as Elephantiasis above			1
	swollen legs	0.111	as Elephantiasis above	-	-	2
**Don't know / Doctor not disclosed**	doctor has not disclosed	NA		-	-	1
	don't know	NA		-	-	1
	don't know	NA		-	-	4
**Didn't want to disclose**		NA		-	-	1
**Total cases**				**6**	**0**	**259**
No health problems				20	24	0
**Total Respondents**				**26**	**24**	**259**
