# Supplementary material for: Development, reliability and validity of the Chichewa WHOQOL-BREF in adults in lilongwe, Malawi
Source: BMC Res Notes. 2012 Jul 3;5:346. doi: 10.1186/1756-0500-5-346 (PMC3483688; doi:10.1186/1756-0500-5-346)
Supplement: Additional file 3 — Table S1. Frequency of each response to each of the questions of the WHOQOL-BREF. [file 1756-0500-5-346-S3.pdf]

Table 2. Frequency of each response to each of the questions of the WHOQoL-BREF

	Survey Question	Response[1]											Distribution[2]
		1 = Very poor		2 = Poor		3 = Neither poor nor good		4 = Good		5 = Very good		missing data	
		n	%	n	%	n	%	n	%	n	%	n	
1	How would you rate your quality of life?	12	4%	26	9%	74	25%	139	46%	49	16%	0	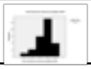
		1 = Very dissatisfied		2 = Dissatisfied		3 = Neither satisfied or dissatisfied		4 = Satisfied		5 = Very satisfied		missing data	
		n	%	n	%	n	%	n	%	n	%	n	
2	How satisfied are you with your health?	6	2%	55	18%	92	31%	116	39%	31	10%	0	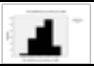
		1 = An extreme amount		2 = Very much		3 = A moderate amount		4 = A little		5 = Not at all		missing data	
		n	%	n	%	n	%	n	%	n	%	n	
3	To what extent do you feel that (physical) pain prevents you from doing what you need to do?	14	5%	54	18%	61	20%	125	42%	46	15%	0	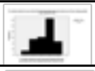
4	How much do you need any medical treatment to function in your daily life?	26	9%	64	21%	71	24%	88	29%	51	17%	0	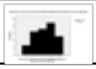
		1 = Not at all		2 = A little		3 = A moderate amount		4 = Very much		5 = An extreme amount		missing data	
		n	%	n	%	n	%	n	%	n	%	n	
5	How much do you enjoy life?	0	0%	24	8%	80	27%	168	56%	28	9%	0	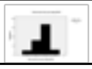
6	To what extent do you feel your life to be meaningful?	2	1%	11	4%	28	9%	147	50%	109	37%	3	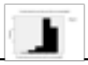
		1 = Not at all		2 = A little		3 = A moderate amount		4 = Very much		5 = Extremely		missing data	
		n	%	n	%	n	%	n	%	n	%	n	
7	How well are you able to concentrate?	6	2%	47	16%	76	25%	118	40%	52	17%	1	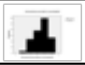
8	How safe do you feel in your daily life?	8	3%	24	8%	78	26%	123	41%	67	22%	0	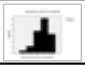
9	How healthy is your physical environment?	8	3%	35	12%	69	23%	137	46%	51	17%	0	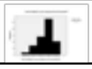
		1 = Not at all		2 = A little		3 = Moderately		4 = Mostly		5 = Completely		missing data	
		n	%	n	%	n	%	n	%	n	%	n	
10	Do you have enough energy for everyday life?	18	6%	88	29%	82	27%	80	27%	32	11%	0	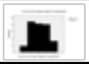
11	Are you able to accept your bodily appearance?	5	2%	6	2%	33	11%	151	51%	99	34%	6	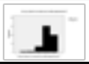
12	Have you enough money to meet your needs?	26	9%	59	20%	114	38%	91	30%	10	3%	0	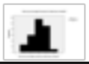
13	How available to you is the information that you need in your day-to-day life?	19	6%	53	18%	94	31%	113	38%	21	7%	0	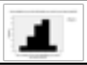
14	To what extent do you have the opportunity for leisure activities?	30	10%	58	19%	83	28%	107	36%	21	7%	1	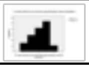
27	To what extent do you have enough food to eat?	7	2%	21	7%	73	24%	164	55%	35	12%	0	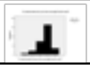

continued on next page

Table 2 continued

	Survey Question	Response[1]											Distribution[2]
		1 = Very poor		2 = Poor		3 = Neither poor nor good		4 = Good		5 = Very good		missing data	
		n	%	n	%	n	%	n	%	n	%	n	
		1 = Very poor		2 = Poor		3 = Neither poor nor good		4 = Good		5 = Very good		missing data	
		n	%	n	%	n	%	n	%	n	%	n	
15	How well are you able to get around?	16	5%	52	18%	78	26%	108	37%	41	14%	5	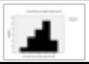
		1 = Very dissatisfied		2 = Dissatisfied		3 = Neither satisfied or dissatisfied		4 = Satisfied		5 = Very satisfied		missing data	
		n	%	n	%	n	%	n	%	n	%	n	
16	How satisfied are you with your sleep?	7	2%	38	13%	64	21%	154	51%	37	12%	0	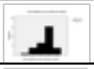
17	How satisfied are you with your ability to perform your daily living activities?	20	7%	52	17%	67	22%	103	34%	58	19%	0	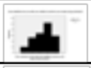
18	How satisfied are you with your capacity for work?	20	7%	44	15%	67	22%	99	33%	70	23%	0	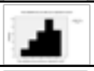
19	How satisfied are you with yourself?	3	1%	9	3%	37	12%	152	51%	98	33%	1	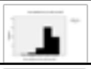
20	How satisfied are you with your personal relationships?	1	0%	18	6%	49	16%	153	51%	79	26%	0	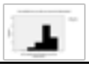
21	How satisfied are you with your sex life?	10	5%	18	9%	39	20%	71	37%	54	28%	108	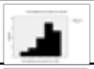
22	How satisfied are you with the support you get from your friends?	7	2%	20	7%	62	21%	171	57%	40	13%	0	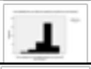
23	How satisfied are you with the conditions of your living place?	23	8%	54	18%	79	26%	121	40%	23	8%	0	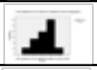
24	How satisfied are you with your access to health services?	3	1%	48	16%	105	35%	125	42%	19	6%	0	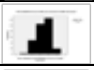
25	How satisfied are you with your transport?	13	4%	62	21%	122	41%	89	30%	14	5%	0	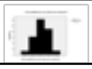
		1 = Always		2 = Very often		3 = Quite often		4 = Seldom		5 = Never		missing data	
		n	%	n	%	n	%	n	%	n	%	n	
26	How often do you have negative feelings such as blue mood, despair, anxiety, depression?	5	2%	19	6%	52	17%	146	49%	78	26%	0	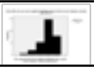

[1]Percentages are of those answering the question. Note that only question 21 has a significant number of non-responders.

[2]A visual representation of the range of responses is provided for each question. The x axis of each graph goes from 1 to 5 from left to right. The y axis gives the frequency of each response (note that the scale of the y axis varies between questions as the some questions have a more skewed distribution of responses than others). Distributions skewed further towards the right are good for all questions. Please note that questions 3, 4 and 26 have been recoded (reversed: 1=5; 2=4; 3=3; 4=2; 5=1) in the database so that 1 is bad and 5 is good to match the other questions and to calculate the overall domain scores.
